# Effects of Botanical Variation, Plant Part, and Extraction Technique on Mitragynine and 7-Hydroxymitragynine in Kratom (*Mitragyna speciosa*) Extracts Quantified by a Validated HPLC–DAD Method

**DOI:** 10.3390/molecules31132241

**Published:** 2026-06-25

**Authors:** Wantanwa Krongrawa, Juthaporn Ponphaiboon, Jariya Koedruen, Chonlakan Samran, Sontaya Limmatvapirat, Chutima Limmatvapirat

**Affiliations:** 1 Natural Products Research Center (NPRC), Faculty of Pharmacy, Silpakorn University, Nakhon Pathom 73000, Thailand; krongrawa_w@su.ac.th (W.K.); augusto_sc@hotmail.co.th (J.P.); koedruen_j@su.ac.th (J.K.); limmatvapirat_s@su.ac.th (S.L.); 2 Pharmaceutical Intellectual Center “Prachote Plengwittaya”, Faculty of Pharmacy, Silpakorn University, Nakhon Pathom 73000, Thailand; 3 Division of Industrial Pharmacy, Faculty of Pharmacy, Silpakorn University, Nakhon Pathom 73000, Thailand; 4 Siriraj Academic Center of Geriatric Medicine, Faculty of Medicine Siriraj Hospital, Mahidol University, Samut Sakhon 74000, Thailand; chonlakan.samran@gmail.com

**Keywords:** *Mitragyna speciosa*, mitragynine, 7-hydroxymitragynine, phytochemical variation

## Abstract

*Mitragyna speciosa* (kratom) is a traditionally consumed botanical rich in the bioactive alkaloids mitragynine (MG) and 7-hydroxymitragynine (7OH-MG). This study evaluated the influence of botanical variation, plant part, geographical origin, and extraction technique on alkaloid composition and extraction yield. A validated HPLC–DAD method was applied to quantify MG and 7OH-MG in leaf and shoot extracts. Ethanol-based ultrasound-assisted extraction (UAE) and microwave-assisted extraction (MAE) were used for samples from northern and southern Thailand. Significant compositional differences were observed. Green-veined leaves contained higher MG levels than red-veined leaves. Northern samples showed higher extraction yields and MG contents, whereas southern samples exhibited relatively higher 7OH-MG levels. Differences between UAE and MAE were minimal. Notably, 7OH-MG was detected only in leaves, while shoots contained higher MG concentrations despite lower yields. These findings highlight substantial phytochemical variability relevant to quality standardization of kratom-derived products.

## 1. Introduction

*Mitragyna speciosa* (Korth.) Havil., commonly known as kratom, is a tropical tree belonging to the Rubiaceae family that is native to Southeast Asia, including Thailand, Malaysia, Indonesia, and neighboring regions. Kratom leaves have long been traditionally consumed for their stimulant, analgesic, and fatigue-relieving properties and are also used in several traditional medicinal preparations [1]. The pharmacological activities of kratom are primarily attributed to its rich alkaloidal content, which comprises approximately 40 different alkaloids [2]. Among these, mitragynine (MG) is the most abundant and pharmacologically significant psychoactive alkaloid in kratom leaves [3]. MG exhibits both psychostimulant- and opioid-like effects in a dose-dependent manner: at low concentrations, it produces stimulant effects, whereas at higher concentrations, it exerts sedative and analgesic activities [4]. Consequently, kratom has been traditionally used to alleviate opioid withdrawal symptoms associated with heroin and morphine dependence [5]. Another important alkaloid present in kratom leaves is 7-hydroxymitragynine (7OH-MG), which has attracted increasing scientific interest due to its potent analgesic activity [6]. Preclinical studies have demonstrated that 7OH-MG exhibits analgesic potency approximately 40 times greater than MG and 10 times stronger than morphine, whereas MG itself shows lower analgesic activity compared to morphine [7]. Notably, extracts containing both MG and 7OH-MG have been reported to provide effective pain relief while reducing morphine tolerance, highlighting their potential pharmaceutical applications [8].

The contents and relative distribution of MG and 7OH-MG in kratom are highly variable and are influenced by multiple intrinsic and extrinsic factors, including botanical variation (e.g., vein type), geographical origin, plant part, cultivation conditions, seasonal variation, and extraction technique [9]. Previous studies have largely focused on kratom leaf extracts, whereas comparative information regarding alkaloid profiles between different plant tissues, such as leaves and shoots, remains limited [10,11]. Importantly, most previous studies have predominantly focused on mature leaves, while limited information is available regarding the comparative alkaloid composition between leaves and shoots. The lack of systematic investigation into tissue-specific distribution represents a critical research gap that may significantly influence the interpretation of kratom phytochemical variability. Various extraction techniques, such as Soxhlet extraction, maceration, ultrasound-assisted extraction (UAE), microwave-assisted extraction (MAE), flash chromatography, and accelerated solvent extraction (ASE), have previously been employed to prepare kratom leaf extracts [12]. Some analytical and preparative methods have utilized organic solvents such as methanol or chloroform because of their high extraction efficiency for alkaloids. However, ethanol- and hydroalcoholic-based extraction systems have also been widely reported and are considered more suitable for pharmaceutical and nutraceutical applications due to their lower toxicity, regulatory acceptance, and environmental compatibility. Previous studies have demonstrated that ethanol extracts of kratom leaves exhibit superior antinociceptive activity compared with methanolic or ethyl acetate extracts, and contain key phytochemicals such as indole alkaloids and flavonols, including MG, paynantheine, quercetin, and rutin [13]. Moreover, UAE has been reported to yield higher MG contents than MAE and supercritical carbon dioxide extraction (SFE-CO_2_) [14]. From a process efficiency perspective, UAE utilizes acoustic cavitation to enhance mass transfer at relatively low temperatures, whereas MAE accelerates extraction through rapid dielectric heating, resulting in reduced extraction time and energy consumption compared with conventional techniques. These complementary mechanisms support their selection as green and efficient extraction approaches. Nevertheless, systematic evaluations comparing the influence of extraction technique alongside botanical variation and plant part under controlled analytical conditions are still scarce. Therefore, ethanol-based UAE and MAE were selected in the present study as practical extraction approaches for comparative evaluation of kratom alkaloid profiles. The findings of this study are also relevant to the broader international context, as kratom products are increasingly used and regulated in markets such as the United States and Europe. Therefore, the systematic characterization of alkaloid variability in Thai kratom can serve as a scientific reference for global quality standardization and regulatory evaluation of kratom-based products.

Accurate and reliable quantification of MG and 7OH-MG is essential for the quality control, standardization, and pharmaceutical development of kratom extracts. Various chromatographic methods have been developed for kratom alkaloid analysis, including high-performance liquid chromatography coupled with ultraviolet detection (HPLC–UV) [15], high-performance liquid chromatography coupled with diode-array detection (HPLC–DAD) [16,17,18], liquid chromatography–tandem mass spectrometry (LC–MS/MS) [19], and ultra-high-performance liquid chromatography coupled with high-resolution mass spectrometry (UHPLC–HRMS) [20]. These techniques have been widely applied for qualitative screening and quantitative determination of major and minor alkaloids in kratom and related products. In particular, LC–MS/MS methods provide high sensitivity and selectivity for multi-alkaloid quantification, enabling reliable detection of compounds such as MG, 7OH-MG, paynantheine, and speciogynine with excellent linearity and low detection limits, making them suitable for regulatory and forensic applications [19]. Similarly, UHPLC–HRMS profiling studies have shown that commercial kratom products and plant materials generally exhibit comparable alkaloid fingerprints, although substantial quantitative variation exists among individual alkaloids and chemotypes [20]. Large-scale product-based investigations have further demonstrated relatively consistent overall alkaloid patterns in whole-leaf kratom preparations, supporting their use in intake estimation and clinical study design [21]. Nevertheless, most published studies have focused on commercial product characterization, alkaloid profiling, or targeted quantification, while providing limited systematic evaluation of the effects of botanical variation, plant part, and extraction technique under standardized extraction and analytical conditions. In addition, some analytical methods require specialized instrumentation or lack comprehensive validation, which may limit their routine implementation in quality control laboratories. Furthermore, many reported methods rely on specialized LC–MS/MS or HRMS platforms, which may restrict routine application because of high instrumentation and operational costs. Differences in validation depth, analytical sensitivity, and methodological complexity may also affect their suitability for routine pharmaceutical and nutraceutical quality assessment.

Several analytical methods have been reported for the determination of MG alone using HPLC-DAD, employing either gradient or isocratic elution systems with different buffer compositions and detection wavelengths [16,17,18]. However, methods for the simultaneous determination of MG and 7OH-MG using HPLC-DAD remain limited. Although some studies have reported simultaneous analysis using RP-C18 columns and alkaline buffer systems, these methods often lack comprehensive method validation data, including specificity, peak purity assessment, system suitability parameters, and representative HPLC chromatograms. As a result, the accuracy, precision, and reliability of the reported analytical results remain uncertain. More recently, HPLC-based methods using UV or DAD detection have been developed for the determination of MG and 7OH-MG in kratom-related products, including herbal teas [16,17,18,19,20,21,22]. Nevertheless, several of these methods exhibit notable limitations, such as incomplete validation, high limits of detection and quantification, absence of peak purity evaluation, reliance on expensive or specialized columns, complex mobile phase preparation, and increased analytical costs. These drawbacks restrict their practical application in routine quality control laboratories and industrial settings.

Given the pronounced variability of MG and 7OH-MG contents arising from differences in botanical characteristics, plant tissues, and extraction strategies, a robust and fully validated analytical method is essential not only for quality control but also for meaningful comparative interpretation of alkaloid profiles across samples. An ideal method should provide high sensitivity, accuracy, and precision, particularly for 7OH-MG, which is present at low concentrations but exhibits strong pharmacological activity [23,24]. Furthermore, the method should be compatible with commonly available HPLC-DAD instrumentation and comply with international method validation guidelines.

Therefore, the objective of the present study was to develop and validate a reliable HPLC-DAD method for the simultaneous quantification of MG and 7OH-MG in kratom leaf extracts, following the principles of ICH Q2(R1) and AOAC guidelines [25,26,27]. The validated method was subsequently applied to systematically investigate the effects of botanical variation, plant part, and extraction technique on the extraction efficiency and alkaloid composition of kratom extracts. The results of this study are expected to support quality control and standardization of kratom extracts at both laboratory and industrial scales, thereby enhancing confidence in product quality and contributing to the sustainable development of kratom-based pharmaceutical and nutraceutical products.

## 2. Results and Discussion

### 2.1. Method Development for the Simultaneous Determination of MG and 7OH-MG

Accurate and reliable quantitative determination of MG and 7OH-MG in kratom extracts is essential for research, quality control, and safety evaluation, as both alkaloids exert pharmacological effects on the central nervous system and variations in their concentrations may influence efficacy and safety. However, kratom extracts possess a complex matrix containing numerous alkaloids and other phytochemicals that may interfere with chromatographic analysis. Therefore, the development of a selective, accurate, and precise analytical method compliant with internationally accepted guidelines is critically required to ensure reliable quantification of MG and 7OH-MG.

#### 2.1.1. Separation Using Gradient Condition 1

Initially, the chromatographic conditions previously reported by Norliana Izzati et al. (2021) were applied to evaluate their suitability for the simultaneous separation of MG and 7OH-MG in kratom leaf extracts [16]. The analysis was performed using a HPLC system (Agilent InfinityLab LC Series 1220 Infinity II LC System, Serial No. DECAH01177, Santa Clara, CA, USA) equipped with a DAD and operated with OpenLAB CDS ChemStation software (version C.01.10). Chromatographic separation was carried out on a Luna^®^ 5 µm C18(2) 100 Å LC column (250 × 4.6 mm, Serial No. H21-301326; Phenomenex, Torrance, CA, USA) at a column temperature of 25 ± 1 °C. UV detection was set at 226 nm, with a flow rate of 1.0 mL/min, an injection volume of 10 µL, and a total run time of 25 min. Gradient elution was performed using 5.0 mM ammonium bicarbonate buffer (pH 9.50) as solvent A and acetonitrile as solvent B. The mobile phase composition was initially set at 20% A and 80% B, gradually increased to 30% A at 5 min and 60% A at 10 min, then further increased to 80% A and maintained until 20 min before returning to the initial composition for column re-equilibration (Table 1).

Under these conditions, multiple small peaks originating from the kratom extract matrix were observed to co-elute with the MG and 7OH-MG peaks, particularly within the retention time window of 10–15 min. This co-elution resulted in inadequate resolution and compromised peak purity, indicating that gradient condition 1 was not suitable for the selective and reliable quantification of MG and 7OH-MG in kratom extracts. Consequently, further optimization of the mobile phase composition and gradient profile was required.

#### 2.1.2. Separation Using Gradient Condition 2

Due to the poor separation achieved under gradient condition 1, the chromatographic conditions were modified based on the method reported by Karunakaran et al., while the same C18 column as that used in gradient condition 1 was retained [28]. Separation was performed at a column temperature of 20 ± 1 °C with UV detection at 226 nm (bandwidth 4 nm), a flow rate of 1.5 mL/min, an injection volume of 5 µL, and a total run time of 60 min. The mobile phase consisted of 0.1% formic acid in water (solvent A) and acetonitrile (solvent B), applied using a gradient program starting at 70% A and 30% B. The proportion of solvent A was gradually decreased to 30% at 25 min and maintained until 35 min, followed by a further decrease to 0% A at 40 min and held until 50 min. The initial mobile phase composition was then restored at 55 min and maintained until the end of the run for column re-equilibration (Table 1).

Chromatograms obtained under gradient condition 2 exhibited an unstable baseline with multiple interfering peaks. Although the MG peak could be clearly detected and met system suitability criteria in terms of symmetry factor (≤2.0), resolution (≥2.0), and theoretical plates (>2000), the peak corresponding to 7OH-MG could not be clearly identified due to severe peak overlap and matrix interference [27]. Therefore, gradient condition 2 was deemed unsuitable for the simultaneous quantification of MG and 7OH-MG.

#### 2.1.3. Separation Using Gradient Condition 3

Since gradient condition 2 provided acceptable separation of MG but failed to resolve 7OH-MG as a distinct peak, further method optimization was undertaken by replacing formic acid with 0.1% trifluoroacetic acid (TFA) in the aqueous mobile phase. The use of TFA was intended to minimize interactions between the basic nitrogen atoms of kratom alkaloids, including MG and 7OH-MG, and residual silanol groups on the C18 stationary phase, which can cause peak tailing and reduced chromatographic performance. By suppressing these secondary interactions, TFA improved peak symmetry and chromatographic resolution. Because the objective of this study was to develop a robust HPLC–DAD method for routine quality-control applications, chromatographic optimization was primarily based on peak symmetry, resolution, baseline separation, and system suitability criteria. All other chromatographic parameters, including column temperature, detection wavelength, flow rate, injection volume, and total run time, were kept unchanged. Gradient elution was carried out using 0.1% TFA in water (solvent A) and acetonitrile (solvent B), beginning at 70% A and 30% B. The proportion of solvent A was gradually reduced to 30% at 25 min and maintained until 35 min, followed by a further decrease to 0% A at 40 min, which was held until 50 min. The mobile phase was then returned to the initial composition at 55 min and maintained until 60 min to re-equilibrate the column (Table 1).

**Figure 1:**
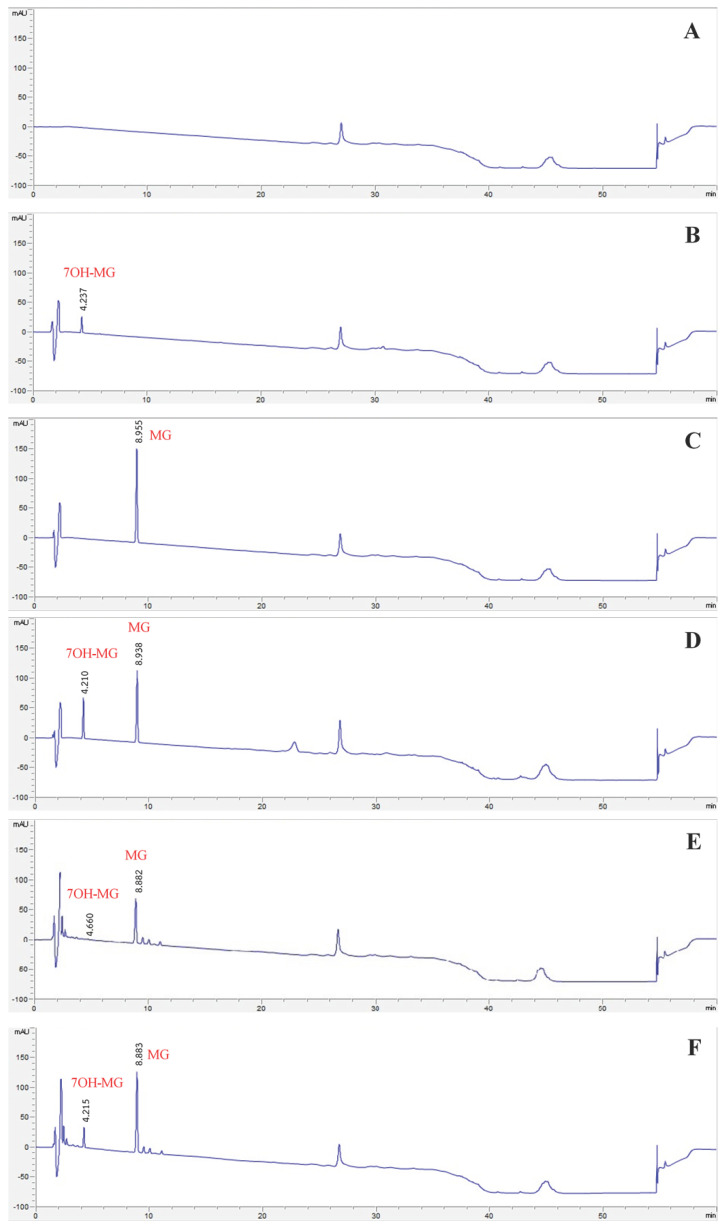
HPLC chromatograms obtained under gradient condition 3 using a Luna^®^ 5 µm C18(2) 100 Å LC column (250 × 4.6 mm) at a column temperature of 25 ± 1 °C with UV detection at 226 nm: (**A**) blank; (**B**) 7-hydroxymitragynine (7OH-MG); (**C**) mitragynine (MG); (**D**) mixed standard of MG and 7OH-MG (MixSTD); (**E**) kratom leaf extract; and (**F**) spiked extract, demonstrating clear peak separation and absence of matrix interference.

Under gradient condition 3, both 7OH-MG and MG were clearly separated and eluted as well-defined, symmetrical peaks with a stable baseline and no observable matrix interference (Figure 1). The retention times, peak areas, symmetry factors, theoretical plate numbers, and resolution values for both analytes satisfied the predefined acceptance criteria, confirming the suitability of this condition for their simultaneous determination in kratom leaf extracts [27].

**Figure 2:**
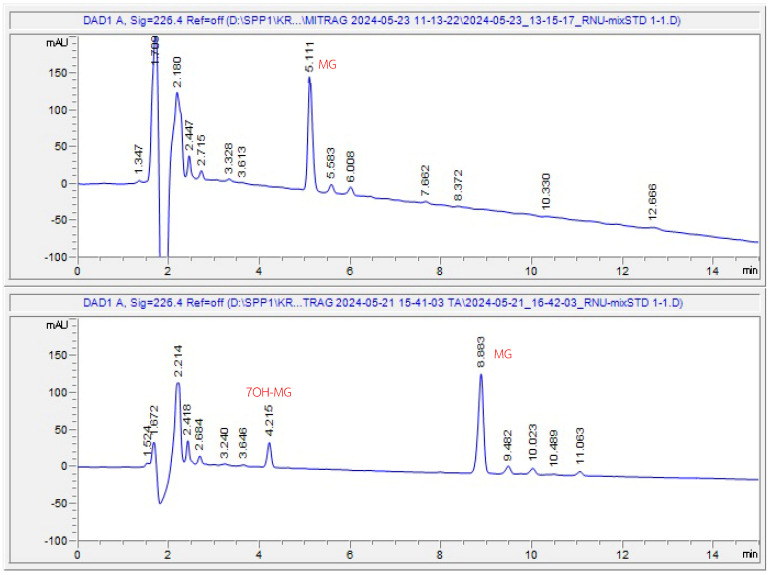
Comparison of HPLC chromatograms of spiked kratom extracts obtained under gradient condition 2 (upper chromatogram) and gradient condition 3 (lower chromatogram), demonstrating the improved separation efficiency, enhanced peak purity, and reduced matrix interference achieved under gradient condition 3.

A direct comparison between chromatograms obtained under gradient conditions 2 and 3 further demonstrated the superior separation efficiency and improved peak purity achieved with the use of 0.1% TFA (Figure 2). Based on these results, gradient condition 3 was selected as the basis for subsequent method refinement. Notably, as both analytes eluted relatively early under these conditions, further optimization focusing on shortening the run time, reducing solvent consumption, and enhancing early peak resolution was considered feasible.

#### 2.1.4. Optimization Using Gradient Conditions 4 and 5

To further enhance separation efficiency while reducing analysis time and solvent consumption, gradient conditions 4 and 5 were developed by modifying the gradient program, flow rate, and run time, while maintaining the mobile phase composition of 0.1% TFA in water and acetonitrile (Table 1). A comparative evaluation of flow rates at 1.0 mL/min (condition 4) and 1.5 mL/min (condition 5) revealed that, although condition 4 yielded higher theoretical plate numbers, it was associated with increased peak asymmetry and, in certain cases, resolution values below the acceptance criterion (≥2.0) (Table 2).

**Figure 3:**
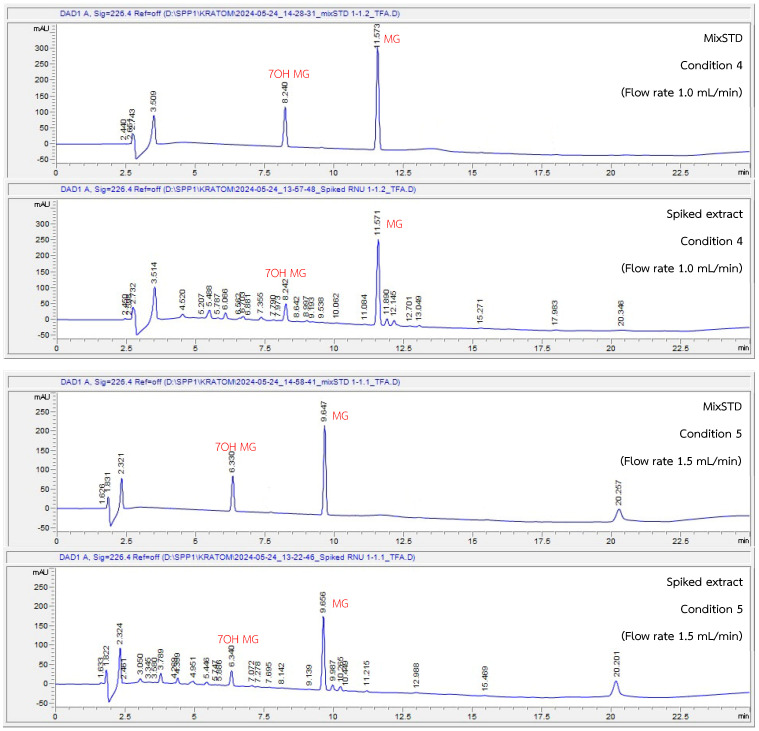
Comparison of HPLC chromatograms obtained at flow rates of 1.0 mL/min (gradient condition 4) and 1.5 mL/min (gradient condition 5) for the analysis of 7-hydroxymitragynine (7OH-MG) and mitragynine (MG) in mixed standard solutions (MixSTD) and spiked kratom leaf extracts, demonstrating the improved peak shape and resolution achieved under gradient condition 5.

In contrast, condition 5 produced well-defined and more symmetrical peaks with satisfactory resolution and theoretical plate numbers for both MG and 7OH-MG. Peak purity analysis based on diode-array spectral evaluation confirmed that the chromatographic peaks of both analytes were spectrally homogeneous under condition 5, indicating the absence of co-eluting interferences. Representative chromatograms illustrating the improved separation achieved under condition 5 compared with condition 4 are shown in Figure 3.

Overall, gradient condition 5 was identified as the most suitable condition for the simultaneous determination of MG and 7OH-MG, as all system suitability parameters, including resolution, peak symmetry, theoretical plate number, and peak purity, consistently met the predefined acceptance criteria.

#### 2.1.5. Optimization Using Gradient Condition 6

Based on the results obtained from the previous experiments, a flow rate of 1.5 mL/min (gradient condition 5) was considered suitable for the simultaneous determination of 7OH-MG and MG using HPLC-DAD. However, during repeated analyses of kratom leaf extract samples, additional late-eluting peaks corresponding to low-polarity components were consistently observed after 15 min. These compounds tended to accumulate on the column, leading to a gradual increase in column backpressure and potentially compromising the long-term separation performance and robustness of the method.

To address this issue, gradient condition 6 was developed (Table 1) by modifying the late stage of the gradient elution program. Specifically, the proportion of acetonitrile was increased from 70% to 100% between 16 and 20 min to more effectively elute low-polarity, strongly retained compounds from the column. Subsequently, the mobile phase composition was returned to the initial conditions between 22.5 and 25 min to allow proper column re-equilibration. Importantly, this modification did not affect the separation efficiency of 7OH-MG and MG, as both analytes eluted before 15 min.

**Figure 4:**
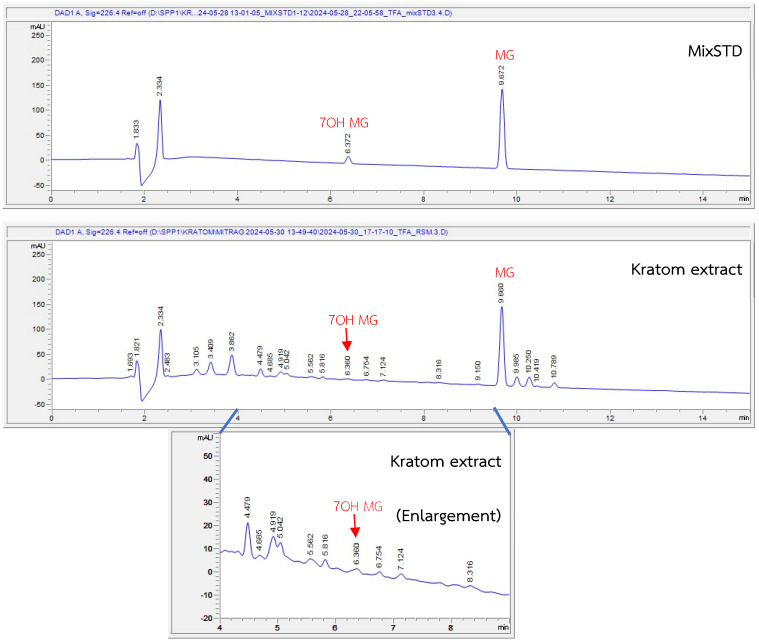
HPLC chromatograms obtained under gradient condition 6 showing (**top**) the mixed standard solution of 7-hydroxymitragynine (7OH-MG) and mitragynine (MG) (MixSTD), (**middle**) kratom extract, and (**bottom**) an enlarged chromatogram highlighting the 7OH-MG peak. Peak identity was confirmed by retention time matching with reference standards and diode-array peak purity assessment.

Based on these findings, gradient condition 6, employing a flow rate of 1.5 mL/min and an enhanced column-cleaning step, was selected as the optimal HPLC condition for the quantitative analysis of 7OH-MG and MG in kratom extracts by HPLC-DAD. A comparative analysis of mixed standard solutions (MixSTD) containing 7OH-MG, MG and kratom leaf extract samples was performed under these conditions. The resulting HPLC chromatograms (Figure 4) demonstrated clear and well-resolved peaks for both analytes, confirming the suitability of gradient condition 6 for their simultaneous determination.

Under gradient condition 6, both analytes exhibited acceptable chromatographic performance, with symmetry factors ≤ 2.0 and resolution values ≥ 2.0. Peak identity was confirmed by comparison with authentic reference standards based on retention time and diode-array spectral matching. Furthermore, peak purity values exceeding 999.00 indicated the absence of significant co-eluting interferences, supporting the specificity of the method and the adequate resolution of 7OH-MG and MG under the optimized chromatographic conditions.

### 2.2. Method Validation for the Simultaneous Determination of MG and 7OH-MG

During method development for the separation and quantitative determination of MG and 7OH-MG in kratom extracts using HPLC–DAD, chromatographic conditions were found to significantly influence separation efficiency, peak shape, baseline stability, and analysis time. Among the evaluated conditions, HPLC condition 6, employing a mobile phase flow rate of 1.5 mL/min with gradient elution, provided clear and reproducible separation of MG and 7OH-MG with symmetrical peak shapes and an efficient analysis time. Therefore, this condition was selected as the optimal analytical condition for subsequent method validation.

For method development and validation, a representative kratom extract was prepared from composite aerial plant material consisting of both leaves and shoots collected and processed together. Therefore, the validation matrix contained constituents originating from both plant tissues and was considered representative of the kratom samples subsequently analyzed in this study. For clarity, the term “kratom extract” is used throughout the validation section to reflect the composition of the matrix employed during method development and validation.

Although adequate chromatographic separation is essential, it is insufficient for reliable quantitative analysis. Method validation is required to ensure that the analytical procedure provides accurate, precise, specific, and reproducible results in accordance with internationally accepted validation criteria. Accordingly, the present study focused on validating the HPLC–DAD method for the simultaneous determination of MG and 7OH-MG in kratom extracts using HPLC condition 6, in order to confirm its suitability for quantitative analysis and quality control applications.

#### 2.2.1. System Suitability

System suitability was evaluated to verify the adequacy of the optimized HPLC-DAD conditions for the simultaneous analysis of MG and 7OH-MG in kratom extracts. A mixed standard solution containing MG (48.0 µg/mL) and 7OH-MG (12.0 µg/mL), corresponding to the mid-point of the linearity range, was injected consecutively nine times. The %RSD of peak areas was calculated, and key chromatographic parameters, including symmetry factor, resolution, and theoretical plate number (N), were assessed against predefined acceptance criteria [27].

As summarized in Table 3, both analytes demonstrated excellent repeatability, with %RSD values of peak areas below 2.0%. The symmetry factors were close to unity and well within the acceptance limit (≤2.0), indicating symmetrical peak shapes. In addition, high-resolution values (≥2.0) and large theoretical plate numbers (>2000) were consistently obtained for both MG and 7OH-MG, reflecting adequate separation efficiency and column performance. Overall, all evaluated parameters complied with the system suitability criteria, confirming that the developed HPLC method was suitable for subsequent quantitative analysis [27].

#### 2.2.2. Specificity

Following the selection of HPLC condition 6 as the optimized analytical condition based on chromatographic performance and separation efficiency, method validation was conducted to confirm the suitability of the method for quantitative analysis. As part of this validation process, specificity was evaluated to assess the ability of the developed HPLC-DAD method to unequivocally separate and identify MG and 7OH-MG in the presence of potential interferences from the kratom extract matrix. Specificity was assessed by analyzing a mixed standard solution of MG and 7OH-MG, kratom extract (1 mg/mL), and spiked samples. The spiked samples were prepared by mixing 0.5 mL of kratom extract (2 mg/mL) with 0.5 mL of a mixed standard solution containing MG (64 µg/mL) and 7OH-MG (16 µg/mL). Chromatographic parameters, including retention time, resolution, and peak purity, were compared among the mixed standard, extract, and spiked samples to evaluate method specificity [25,26].

Specificity was evaluated by comparing the chromatographic behavior of MG and 7OH-MG in MixSTD, kratom extract, and spiked samples. The assessment was based on retention time consistency, chromatographic resolution, and peak purity obtained from DAD spectral analysis. For MG, the retention times obtained from MixSTD, extract, and spiked samples were highly consistent, with mean values of 9.72 ± 0.04 min. Adequate chromatographic resolution (≥0.95) was achieved in all sample types, and peak purity values exceeded the acceptance criterion (>999.00), indicating the absence of co-eluting interferences from the extract matrix. Similarly, 7OH-MG showed consistent retention times across MixSTD, extract, and spiked samples, with a mean retention time of 6.37 ± 0.01 min. The resolution values confirmed effective separation of 7OH-MG from adjacent peaks in all matrices. In addition, peak purity values obtained by DAD spectral analysis were higher than the predefined acceptance threshold, demonstrating that the detected peaks corresponded exclusively to 7OH-MG without spectral interference. Overall, the consistent retention times, satisfactory resolution, and high peak purity values for both analytes across all tested matrices confirm that the optimized HPLC-DAD method is sufficiently specific for the simultaneous identification and quantification of MG and 7OH-MG in kratom extracts. The detailed specificity evaluation results are summarized in Table 4.

#### 2.2.3. Accuracy

The accuracy of the developed HPLC–DAD method was evaluated using the standard addition (spiking) approach to assess its ability to accurately quantify MG and 7OH-MG in the kratom extract matrix. Spiked samples were prepared by fortifying kratom extract (2 mg/mL) with MixSTD of MG and 7OH-MG at three concentration levels corresponding to 75%, 100%, and 125% of the nominal concentration, as recommended by validation guidelines [25,27]. The final concentrations were 24, 32, and 40 µg/mL for MG and 6, 8, and 10 µg/mL for 7OH-MG, respectively. Each concentration level was analyzed in triplicate under the optimized HPLC–DAD conditions. Method accuracy was evaluated based on percentage recovery (%recovery), while precision at each level was assessed using the %RSD.

The accuracy results are summarized in Table 5. The %recovery values for MG ranged from 107.68% to 109.47%, whereas those for 7OH-MG ranged from 98.28% to 108.24%. All recovery values were within the predefined acceptance range of 80–110% [25]. In addition, the %RSD values for both analytes at all concentration levels were below 2.0%, indicating good repeatability of the method [25].

Overall, the accuracy study demonstrates that the proposed HPLC–DAD method provides accurate and reliable quantification of MG and 7OH-MG in the kratom extract matrix. The consistent recovery values across all concentration levels indicate negligible matrix interference, while the low %RSD values confirm the good precision of the analytical procedure. These results support the suitability of the developed method for quantitative analysis and routine quality control of kratom extracts.

#### 2.2.4. Precision

The precision of the developed HPLC–DAD method was evaluated to assess the repeatability and reproducibility of the analytical procedure under the optimized conditions. Precision was expressed as the %RSD of replicate measurements, with an acceptance criterion of %RSD ≤ 2.0%. In addition, method precision was further evaluated using the Horwitz ratio (HorRat), with acceptable values ranging from 0.5 to 2.0, in accordance with AOAC guidelines [22]. Precision was investigated at three concentration levels (75%, 100%, and 125% of the nominal concentration) using spiked kratom extract samples and was assessed under two conditions: intra-day precision and inter-day precision. Intra-day precision was determined by analyzing spiked samples at three concentration levels, with six replicate injections per level, within a single analytical day. The %RSD and HorRat values were calculated for each analyte, and the results were considered acceptable when the %RSD did not exceed 2.0%. Inter-day precision was evaluated by analyzing the same spiked samples at three concentration levels over three consecutive days, with three replicate injections per level per day. The resulting data were used to calculate %RSD and HorRat values, applying the same acceptance criteria as those used for intra-day precision.

The HorRat value was used as an indicator of the adequacy of method precision by comparing the experimentally observed variability with the predicted variability based on the Horwitz equation, which is widely accepted in AOAC validation guidelines [26]. The HorRat was calculated according to Equation (1)

HorRat = Observed %RSD/Predicted %RSD(1)

where the observed %RSD represents the experimentally determined relative standard deviation obtained from the precision study. The predicted %RSD was calculated using the Horwitz equation (Equation (2))

Predicted %RSD = 2^(1 − 0.5 log C)^ (2)

where C represents the analyte concentration expressed as a mass fraction (g/g). Based on the concentration levels evaluated in this study, the corresponding mass fractions were within the range typically associated with predicted %RSD values of approximately 9–10% according to the Horwitz equation. Because the calculated predicted %RSD values varied only slightly across the concentration range studied, a value of 9.5% was used as a constant predicted %RSD for HorRat calculations. This approach was considered appropriate since minor differences in analyte concentration had a negligible effect on the predicted variability.

The precision results for MG and 7OH-MG are summarized in Table 6. For both analytes, the %RSD values obtained from the intra-day and inter-day precision studies were consistently below 2.0%, meeting the predefined acceptance criteria. These findings demonstrate good repeatability and intermediate precision of the method under identical analytical conditions as well as across different days. Although the generally accepted HorRat range is 0.5–2.0, the HorRat values obtained in this study were consistently below 0.5, indicating a level of precision higher than that predicted by the Horwitz equation. Such low HorRat values are commonly observed in well-controlled single-laboratory validations employing modern instrumental analytical techniques. The consistently low HorRat values for both MG and 7OH-MG across all concentration levels indicate that the observed precision of the method was substantially better than the theoretical expectation. Importantly, despite being lower than 0.5, these HorRat values are still considered acceptable according to AOAC guidelines [26].

Overall, the precision study confirms that the developed HPLC–DAD method exhibits high precision, consistent analytical performance, and excellent reproducibility. The method is therefore suitable for quantitative determination of MG and 7OH-MG in kratom extracts for both research applications and routine quality control.

#### 2.2.5. Linearity and Range

**Figure 5:**
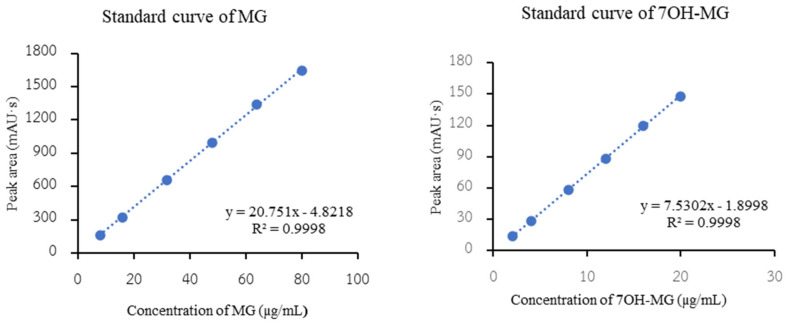
Calibration curves showing the linear relationship between peak area and concentration for mitragynine (MG) and 7-hydroxymitragynine (7OH-MG) obtained by HPLC-DAD under optimized chromatographic condition 6. Good linearity was observed over the validated concentration ranges.

Linearity of the HPLC–DAD method was evaluated using mixed standard solutions of MG and 7OH-MG prepared at multiple concentration levels. The concentration ranges investigated were 8.0–80.0 µg/mL for MG and 2.0–20.0 µg/mL for 7OH-MG. Calibration curves were constructed by plotting the peak area against the corresponding analyte concentration for each compound. Good linear relationships were obtained for both MG and 7OH-MG over the studied concentration ranges, as evidenced by the linear regression analysis (Figure 5). The calibration equations were y = 20.751x − 4.8218 (R^2^ = 0.9998) for MG and y = 7.5302x − 1.8998 (R^2^ = 0.9998) for 7OH-MG. Linearity was evaluated according to ICH Q2(R1) guidelines [21] using linear regression analysis, and the obtained correlation coefficients demonstrated excellent linearity throughout the validated concentration ranges. The results demonstrate that the detector response was directly proportional to analyte concentration within the tested ranges, confirming the linearity of the method.

The analytical range of the method was subsequently established by considering not only the linearity results but also the accuracy and precision data obtained from spiked kratom leaf extract samples. Accuracy and precision were evaluated at three concentration levels corresponding to 75%, 100%, and 125% of the nominal concentration, which fell within the linearity ranges of the calibration curves. The acceptable recovery values (80–110%) and low %RSD values (≤2.0%) obtained at these levels, as presented in the accuracy and precision sections, further support the suitability of the selected concentration ranges for quantitative analysis. Potential matrix effects were indirectly assessed through these spiked-sample accuracy and precision studies, which demonstrated satisfactory method performance in the kratom extract matrix and provided indirect evidence that matrix interference was minimal under the optimized chromatographic conditions.

Based on the combined evaluation of linearity, accuracy, and precision, the working ranges of the method were defined as 8.0–80.0 µg/mL for MG and 2.0–20.0 µg/mL for 7OH-MG. These ranges are therefore considered appropriate for the reliable quantitative determination of MG and 7OH-MG in kratom extracts using the proposed HPLC–DAD method.

#### 2.2.6. Sensitivity

Method sensitivity was evaluated in terms of the limit of detection (LOD) and the limit of quantitation (LOQ), which describe the ability of the method to detect and quantify analytes at low concentration levels. The LOD and LOQ values were calculated from the linear regression parameters of the calibration curves in accordance with the ICH Q2(R1) guideline [25].

The LOD, defined as the lowest detectable concentration of the analyte, and the LOQ, defined as the lowest concentration that can be quantified with acceptable accuracy and precision, were calculated using Equations (3) and (4), respectively

LOD = 3.3 × (SD_intercept_/slope) (3)

LOQ = 10 × (SD_intercept_/slope) (4)

where SD_intercept_ is the standard deviation of the intercept of the calibration curve and slope is the slope of the corresponding regression equation.

Based on these calculations, the LOD and LOQ values for MG were 1.56 and 4.72 µg/mL, respectively, whereas those for 7OH-MG were 0.64 and 1.94 µg/mL, respectively (Table 7). These values were calculated as statistical estimates of method sensitivity according to ICH Q2(R1) [25] and do not represent experimentally verified calibration points. Therefore, method linearity was confirmed only within the validated concentration ranges described in Section 2.2.5, while the calculated LOD and LOQ values indicate the estimated detection and quantification capability of the method. The low LOD and LOQ values obtained demonstrate that the developed HPLC–DAD method possesses sufficient analytical sensitivity for the determination of MG and 7OH-MG in kratom extracts.

#### 2.2.7. Robustness

The robustness of the developed HPLC–DAD method was evaluated by increasing the column temperature by 5 °C from the nominal condition (20 °C to 25 °C), in accordance with the ICH Q2(R1) guideline [25]. Column temperature was selected as a representative robustness parameter because it can significantly influence analyte retention, peak shape, and chromatographic resolution in reversed-phase HPLC systems. In addition, temperature was considered particularly relevant because kratom alkaloids may undergo oxidative or thermal transformation under certain conditions. Therefore, a deliberate temperature variation of +5 °C was applied to assess the ability of the optimized method to maintain acceptable analytical performance under minor operational changes. A mixed standard solution containing MG (48.0 µg/mL) and 7OH-MG (12.0 µg/mL) was analyzed in triplicate under each condition. Robustness was evaluated using mixed standard solutions to assess the intrinsic stability of the chromatographic system independent of sample-matrix variability. The detailed chromatographic data obtained under nominal and elevated temperature conditions are summarized in Table 8. As shown in Table 8, no significant changes were observed in the chromatographic performance of the method following the temperature increase. For both MG and 7OH-MG, the %RSD values of retention time and peak area were ≤2.0%, fulfilling the acceptance criteria for method precision under robustness testing. The peak symmetry factors for both analytes remained ≤2.0, indicating acceptable peak shape under both temperature conditions. In addition, the number of theoretical plates (N) at 25 °C remained greater than 99% of the corresponding values obtained under nominal conditions and exceeded the predefined threshold of ≥80% of nominal performance. The chromatographic resolution (Rs) between 7OH-MG and MG remained well above the minimum acceptance criterion of Rs ≥ 2.0 under both conditions. Although minor variations in resolution were observed following the temperature increase, with maximum percentage changes of 0.10% for MG and 3.50% for 7OH-MG, these variations were considered negligible and remained within acceptable limits [29].

Overall, the HPLC–DAD method developed in this study was fully validated in accordance with ICH Q2(R1) and AOAC guidelines and demonstrated satisfactory system suitability, specificity, linearity, accuracy, precision, sensitivity, and robustness (Table 9). These validation results confirm that the method is reliable and suitable for the quantitative determination of MG and 7OH-MG in kratom extracts, providing a robust analytical foundation for evaluating the effects of botanical variation, plant part, and extraction technique discussed in this work.

### 2.3. Extraction Yield and Alkaloid Profiles of Kratom Leaf Extracts

To clarify the factors governing extraction efficiency and alkaloid composition of kratom, this study systematically examined the effects of botanical variation, geographical origin, and plant part using a validated HPLC–DAD method for the quantitative analysis of MG and 7OH-MG. Leaf extracts obtained from different vein types and regions were compared using UAE and MAE to elucidate the relative influence of intrinsic plant characteristics versus extraction technique. In addition, tissue-specific differences were investigated by comparing leaf- and shoot-derived extracts prepared under identical UAE conditions and controlled particle size, thereby isolating the effect of plant part on alkaloid distribution.

#### 2.3.1. Effect of Vein Type and Geographical Origin on Kratom Leaf Extracts Obtained by UAE and MAE

The percentage yield (% yield), MG content, and 7OH-MG content of kratom leaf extracts obtained from different vein types and geographical regions using UAE and MAE are summarized in Table 10. Overall, the geographical origin of kratom leaves exerted a more pronounced effect on extraction yield than the extraction technique or vein type. Kratom leaf extracts from the northern region exhibited significantly higher % yield than those from the southern region (*p* < 0.05) across both red-veined and green-veined varieties, regardless of the extraction method employed. Northern samples yielded 25.11–25.96% (Group I), whereas southern samples showed markedly lower yields in the range of 17.30–18.43% (Group III). Hang Kang kratom (red-veined) from the northern region (H-N-UAE and H-N-MAE) exhibited intermediate yields (approximately 20%), which were significantly lower than those of other northern samples but remained higher than those of southern samples. When comparing extraction techniques, no statistically significant differences in % yield were observed between UAE and MAE within samples of the same origin and particle size, indicating comparable extraction efficiencies. Although slight differences in particle size were observed among samples (78.32–112.22 μm), all materials were within a relatively fine particle-size range. Under such conditions, the available surface area for solvent penetration and mass transfer was expected to be sufficiently high, thereby minimizing the influence of particle-size variation on extraction performance. Furthermore, the mechanical effects of ultrasonic cavitation during UAE and rapid cell disruption caused by microwave energy during MAE are known to enhance mass transfer and facilitate the release of intracellular constituents. Therefore, the observed differences in extraction yield and alkaloid content are more likely attributable to botanical and geographical factors than to the relatively small variations in particle size.

As presented in Table 10, MG content followed a trend similar to that observed for extraction yield. Northern kratom leaf extracts consistently contained significantly higher MG levels than southern samples for all vein types and extraction methods (*p* < 0.05). The highest MG content was observed in green-veined kratom from the northern region extracted by UAE (G-N-UAE, 58.07 ± 0.06 µg/mg extract), followed by red-veined northern samples (R-N-UAE and R-N-MAE, approximately 57.20–57.57 µg/mg extract). In contrast, southern samples exhibited substantially lower MG contents, ranging from 46.14 to 47.39 µg/mg extract.

Hang Kang kratom from the northern region showed moderate MG contents (H-N-UAE and H-N-MAE, approximately 48.20–48.61 µg/mg extract), which were significantly lower than those of other northern varieties. Green-veined kratom tended to yield slightly higher MG levels than red-veined kratom, particularly in samples from the northern region. UAE produced marginally higher MG contents than MAE in several cases; however, the differences were relatively small.

In contrast to MG, the content of 7OH-MG was significantly higher in extracts obtained from the southern region (*p* < 0.05). Southern samples contained 7OH-MG in the range of 2.33–2.71 µg/mg extract, whereas northern samples exhibited lower levels (1.91–2.59 µg/mg extract) (Table 10). Both red-veined and green-veined kratom from the southern region consistently showed higher 7OH-MG contents than their northern counterparts. In contrast, Hang Kang kratom extracts showed no detectable levels of 7OH-MG under the analytical conditions employed. Differences between UAE and MAE were minor, indicating that the extraction technique was not a dominant factor influencing 7OH-MG content. Although elevated temperatures may promote oxidative transformation of alkaloids, the relatively mild MAE conditions employed in this study (50 °C for 15 min) did not result in a consistent increase in 7OH-MG levels compared with UAE. This observation suggests that thermal conversion of MG to 7OH-MG was limited under the applied extraction conditions. Therefore, the differences in 7OH-MG content observed among samples are more likely attributable to botanical and geographical factors than to temperature-induced oxidation during extraction.

The physical characteristics of kratom leaf extracts obtained using UAE and MAE are summarized in Table 11. Clear differences in extract appearance and texture were observed among kratom varieties and geographical origins, whereas differences between extraction techniques were less pronounced. Extracts from Hang Kang red-veined kratom (HN) exhibited a viscous, sticky consistency with agglomerated masses and a dark greenish-brown color following both UAE and MAE. In contrast, extracts derived from red-veined and green-veined kratom from both northern and southern regions were generally dry, free-flowing, and could be readily ground into fine powders, exhibiting a dark green to brownish-green appearance. Representative photographs illustrating the physical appearance of all extracts obtained by UAE and MAE are provided in Table 11 to support visual comparison.

#### 2.3.2. Comparison of Kratom Extracts Obtained from Leaves and Shoots Using UAE

Table 12 presents the % yield of kratom extracts obtained from shoots and leaves of green vein and red vein kratom using UAE. The plant materials were collected from Nakhon Pathom Province in the central region of Thailand. All samples were dried, ground, and sieved through a single ASTM sieve No. 120 (nominal aperture size 125 µm) to obtain a uniform powder fraction prior to extraction. Particle size distribution analysis was not performed due to limited sample availability. The results showed that extracts derived from kratom leaves exhibited significantly higher extraction yields than those obtained from shoots for both kratom varieties (*p* < 0.05). Among all samples, the red vein leaf extract [R-NP(L)] produced the highest yield (19.77 ± 1.16%), followed by the green vein leaf extract [G-NP(L)] (18.42 ± 0.61%). In contrast, lower yields were observed for extracts prepared from shoots, with yields of 15.65 ± 0.22% for green vein shoots [G-NP(S)] and 14.77 ± 0.46% for red vein shoots [R-NP(S)]. These findings indicate that, under identical UAE conditions and controlled powder size obtained by sieving, the plant part used for extraction plays a crucial role in determining extraction efficiency.

As summarized in Table 12, the MG content of kratom extracts differed significantly according to both kratom variety and plant part (*p* < 0.05). The highest MG content was observed in the green vein shoot extract [G-NP(S)] (79.75 ± 0.07 µg/mg extract). This was followed by the green vein leaf extract [G-NP(L)] (75.22 ± 0.15 µg/mg extract) and the red vein shoot extract [R-NP(S)] (74.53 ± 0.08 µg/mg extract), whereas the lowest MG content was detected in the red vein leaf extract [R-NP(L)] (70.32 ± 0.05 µg/mg extract). Within the same kratom variety, shoot extracts consistently exhibited higher MG contents than the corresponding leaf extracts, indicating that the distribution of MG varies with plant part. Moreover, green vein kratom extracts showed higher MG contents than red vein extracts in both shoots and leaves. As all samples were prepared using identical UAE conditions and a uniform powder fraction obtained by sieving through ASTM sieve No. 120, the observed differences in MG content are attributable primarily to intrinsic varietal and tissue-related factors rather than differences in particle size or extraction parameters.

The distribution of 7OH-MG differed markedly from that of MG. As presented in Table 12, 7OH-MG was detected exclusively in leaf extracts of both kratom varieties, whereas no detectable levels were observed in shoot extracts. Among the leaf samples, the red vein leaf extract [R-NP(L)] exhibited the highest 7OH-MG content (1.22 ± 0.04 µg/mg extract), followed by the green vein leaf extract [G-NP(L)] (0.91 ± 0.03 µg/mg extract). The absence of 7OH-MG in shoot extracts suggests that this alkaloid is preferentially accumulated or formed in mature leaf tissues rather than in actively growing shoots, independent of particle size. It should also be noted that the absence of detectable 7OH-MG in shoots may reflect concentrations below the detection limit (LOD) of the HPLC–DAD method rather than complete absence. Compared with LC–MS/MS-based techniques, HPLC–DAD generally exhibits lower sensitivity, which may limit trace-level detection of minor alkaloids such as 7OH-MG.

Because all samples were prepared using powders with the same average particle size, differences in extraction yield and alkaloid content can be attributed primarily to the plant part and vein type rather than to particle size effects. While leaf powders provided higher extraction yields, shoot extracts exhibited higher MG concentrations, particularly in green vein kratom. These results demonstrate that extraction yield alone does not adequately reflect alkaloid extraction efficiency and highlight the importance of simultaneous evaluation of both yield and target alkaloid content.

The physical appearance of dried kratom leaves and shoots, their corresponding powders, and UAE-derived extracts are summarized in Table 13. Representative images illustrating these physical characteristics are included in the table for visual comparison. Leaf-derived extracts generally appeared as dry, free-flowing powders with a dark greenish-brown coloration, whereas shoot-derived materials exhibited more fibrous characteristics prior to extraction. These physical differences may partially contribute to the observed variations in extraction yield and alkaloid profiles.

Taken together, these results indicate that intrinsic plant-related factors—particularly geographical origin and tissue type—are the primary determinants of extraction yield and alkaloid composition in kratom, whereas vein type and extraction technique exert comparatively minor effects under the conditions employed. Across both UAE and MAE, kratom leaves from the northern region consistently yielded higher extractable mass and MG contents than southern samples, while 7OH-MG was enriched in southern-derived leaves. This opposing regional trend strongly suggests differential regulation of alkaloid biosynthesis and downstream transformation processes rather than extraction-driven selectivity [30,31].

MG is widely regarded as the major biosynthetic indole alkaloid in kratom, whereas 7OH-MG is considered a minor constituent that arises predominantly through oxidative modification of MG [30,32]. The elevated MG levels observed in northern samples may therefore reflect environmental conditions that favor primary alkaloid biosynthesis, such as optimal growth temperature, light exposure, or soil characteristics. The climatic differences between northern and southern Thailand, particularly in terms of rainfall distribution, humidity, and temperature variation, may further contribute to region-specific secondary metabolite biosynthesis patterns. In contrast, the consistently higher 7OH-MG contents detected in southern samples are indicative of enhanced oxidative metabolism or stress-associated transformation pathways, consistent with previous reports linking MG/7OH-MG ratios to environmental and physiological factors [6,30,31,32]. Although 7OH-MG is recognized as a naturally occurring minor alkaloid in kratom, recent studies suggest that part of the detected 7OH-MG may arise through oxidative transformation of MG during plant metabolism, storage, processing, or extraction. Therefore, the higher 7OH-MG levels observed in certain plant tissues or geographical samples may reflect both intrinsic biochemical variation and potential oxidative conversion processes. In addition to alkaloids, kratom leaf extracts may contain co-extracted polyphenols, flavonoids, and other secondary metabolites that could potentially influence the stability and extraction behavior of MG during processing. These matrix components may contribute to oxidation or interaction phenomena under thermal or solvent-assisted extraction conditions, although their specific effects were not individually quantified in the present study.

Vein type contributed only modestly to variation in alkaloid content, with green-veined leaves showing a slight tendency toward higher MG levels. This observation supports the view that vein color alone is not a reliable proxy for alkaloid composition when compared with broader geographic and metabolic determinants. Similarly, the comparable extraction yields and alkaloid profiles obtained by UAE and MAE indicate that, under ethanol-based conditions, both techniques largely reflect the intrinsic chemical profile of the plant material rather than inducing selective enrichment or degradation of MG or 7OH-MG.

Compared with conventional Soxhlet extraction and long-duration maceration, UAE and MAE provide substantially shorter extraction times and lower solvent consumption while maintaining comparable alkaloid recovery. Soxhlet extraction may achieve exhaustive extraction but involves prolonged heating, which may increase the risk of alkaloid degradation or oxidative transformation. ASE has also been reported as a green and efficient alternative for kratom alkaloid extraction under pressurized conditions; however, it requires specialized instrumentation and higher operational costs, which may limit routine laboratory applicability. Under optimized ethanol or hydroalcoholic systems, ASE has been shown to effectively enhance MG yield while maintaining bioactivity, supporting its suitability for phytochemical optimization studies [33]. Under the ethanol-based conditions employed in the present study, UAE and MAE produced comparable alkaloid profiles, suggesting that intrinsic plant characteristics exerted a greater influence on alkaloid distribution than the extraction technique itself. Therefore, while advanced extraction techniques such as ASE may improve efficiency under optimized conditions, UAE and MAE remain practical, scalable, and cost-effective approaches for routine comparative analysis of kratom alkaloids.

From an industrial perspective, both UAE and MAE offer advantages for large-scale kratom extraction due to their relatively short processing times and efficient solvent utilization. However, because MG and 7OH-MG may undergo oxidative or thermal transformation under elevated temperatures, the lower operating temperature employed in UAE may provide an additional advantage for preserving alkaloid integrity during processing. Although MAE can achieve rapid and efficient extraction, careful control of temperature and microwave power is required to minimize the risk of undesirable chemical changes during scale-up. Conversely, UAE is generally associated with lower capital investment, wider industrial availability, and simpler process control, although heat accumulation during prolonged operation of high-power ultrasonic systems should also be monitored. Considering the comparable extraction performance observed in the present study, UAE may represent a more practical and cost-effective approach for industrial production and standardization of kratom extracts, particularly when preservation of native alkaloid composition is a primary objective.

Additional insight into alkaloid localization was provided by the comparison of leaf- and shoot-derived extracts prepared under identical UAE conditions and controlled particle size. Although leaves yielded a greater total extractable mass, shoots consistently exhibited higher MG concentrations, while 7OH-MG was detected exclusively in leaf tissues. This tissue-specific distribution suggests that MG biosynthesis may be more active or concentrated in younger, actively growing tissues, whereas oxidative conversion to 7OH-MG preferentially occurs in mature leaves that are more exposed to light, oxygen, and oxidative enzymes [6,34]. In addition, developmental differences in enzymatic activity may contribute to the absence of detectable 7OH-MG in shoots. Previous studies have demonstrated that MG can undergo cytochrome P450-mediated oxidation to form 7OH-MG in mammalian systems [6]. Although the corresponding pathway has not been confirmed in kratom plants, lower expression or activity of oxidative enzymes in young tissues may limit the formation and accumulation of 7OH-MG during early developmental stages. These findings underscore the importance of plant developmental stage and tissue specialization in shaping kratom alkaloid profiles.

Importantly, the present data demonstrate that extraction yield alone is not a reliable indicator of alkaloid recovery. Rather, meaningful evaluation of kratom extracts requires simultaneous consideration of yield, individual alkaloid content, and their relative ratios, particularly when extracts are intended for pharmacological or functional applications.

This study demonstrates that geographical origin and plant tissue are the dominant factors governing extraction yield and alkaloid composition in kratom, whereas vein type and extraction technique play secondary roles under the conditions examined. Northern kratom leaves were characterized by higher MG contents and extraction yields, while southern samples exhibited elevated levels of 7OH-MG, reflecting region-dependent differences in alkaloid biosynthesis and oxidative transformation. Tissue-specific analysis further revealed preferential enrichment of MG in shoots and exclusive detection of 7OH-MG in leaves, highlighting the influence of plant developmental stage on alkaloid distribution. The validated HPLC–DAD method enabled reliable discrimination of these intrinsic variations and provides a robust analytical framework for the phytochemical characterization and standardization of kratom raw materials.

## 3. Materials and Methods

### 3.1. Chemicals and Reagents

Acetonitrile (HPLC grade; Lot No. J50109) was purchased from Fisher Scientific Korea Ltd. (Seoul, Republic of Korea). Trifluoroacetic acid (TFA; Lot No. 2209093) was obtained from Fisher Scientific UK Ltd. (Leicestershire, UK). Methanol (MeOH, HPLC grade; CAS No. 67-56-1) was supplied by RCI Labscan Limited (Bangkok, Thailand). Ethanol (95% *v*/*v*, Pure Extra Neutral Alcohol; CAS No. 64-17-5) was obtained from the Liquor Distillery Organization (Chachoengsao, Thailand). Mitragynine (MG, 100 µg/mL in methanol; Lot No. FN07262206) and 7-hydroxymitragynine (7OH-MG, 100 µg/mL in methanol containing 0.1 N NH3; Lot No. FN03162211) were purchased from Merck KGaA (Darmstadt, Germany).

### 3.2. Preparation of Kratom Leaf Powder

Different varieties of kratom, including red-veined Hang Kang, red-veined, and green-veined types, were obtained from certified organic cultivation sites in Chiang Rai Province (Northern Thailand) and Nakhon Si Thammarat Province (Southern Thailand). In addition, red-stemmed and green-stemmed kratom samples were collected from an organic farm located in Nakhon Pathom Province (Central Thailand). All samples were collected in December 2024. Voucher specimens (No. 011/2025) were authenticated based on morphological characteristics by Professor Sontaya Limmatvapirat and Associate Professor Chutima Limmatvapirat and deposited at the Faculty of Pharmacy, Silpakorn University, Nakhon Pathom, Thailand.

Mature leaves, defined as leaves located below the third leaf from the apex, were harvested from kratom trees aged at least three years. The collected leaves were thoroughly washed with tap water and rinsed with distilled water to remove surface contaminants. Petioles and major leaf veins, including the midvein and lateral veins, were carefully removed. The leaf laminae were then dried in a hot-air oven at 45 °C until a constant weight was achieved. The dried leaves were subsequently ground into a fine powder. The particle size of the kratom leaf powder was determined, and the powder was stored at −20 °C prior to extraction by UAE or MAE.

### 3.3. Determination of Kratom Leaf Powder Particle Size

The particle size distribution of the kratom leaf powder was determined using an analytical sieve shaker (US standard sieve set, Retsch, Haan, Germany). Briefly, 50 g of each kratom leaf powder sample was placed onto the top sieve of the stacked sieve set. The sieve shaker was operated for 5 min. The weight of each sieve was recorded before and after sieving to determine the amount of powder retained on each sieve. The mean particle size of each kratom leaf powder sample was calculated based on the weight distribution across the sieve fractions.

### 3.4. Extraction of Kratom Leaves

Kratom leaf extraction was performed using either UAE or MAE. For UAE, kratom leaf powder (2 g) was mixed with 20 mL of 95% (*v*/*v*) ethanol, corresponding to a solid-to-solvent ratio of 1:10 (*w*/*v*). The extraction was conducted at 25 °C for 15 min using an ultrasonic bath (Crest Ultrasonics Powersonic Model 230D, Crest Ultrasonics Corporation, Ewing Township, NJ, USA) operating at a frequency of 40 kHz and an output power of 160 W. For MAE, kratom leaf powder (2 g) was mixed with 20 mL of 95% (*v*/*v*) ethanol at a solid-to-solvent ratio of 1:10 (*w*/*v*). The extraction was performed at 50 °C for 15 min using a high-performance microwave digestion system (Ethos One, Milestone Helping Chemists, Sorisole, Italy) with a microwave power of 300 W. The MAE system was equipped with a frequency-stabilized microwave generator operating at 2.45 GHz with adjustable microwave power up to 300 W, enabling controlled dielectric heating during extraction.

After completion of extraction by each technique, the resulting extracts were filtered through Whatman No. 1 filter paper (Cytiva, Marlborough, MA, USA) to separate the liquid extract from plant residues. The filtrates were concentrated by evaporating ethanol using a water bath at 45 °C. The concentrated extracts were subsequently dried by freeze-drying (Freeze Dryer, Model ScanVac CoolSafe 110-4, LaboGene™, Lillerød, Denmark) to obtain dried kratom leaf extracts. The dried extracts were weighed to calculate the extraction yield (% yield) and stored in airtight containers, protected from light, and kept in a cool, dry environment at −20 °C until further analysis by HPLC-DAD. All extraction experiments were performed in triplicate (n = 3), and each sample was analyzed by HPLC in triplicate injections to ensure analytical repeatability and statistical reliability.

### 3.5. Development and Validation of the Analytical Method

Reference standards of MG, 7OH-MG, and the prepared kratom leaf extract were used for the development and validation of the analytical method for quantitative determination of MG and 7OH-MG using HPLC-DAD.

#### 3.5.1. Preparation of the Mobile Phase

The mobile phase was developed based on previous studies, with modifications to the type and ratio of organic solvents as well as pH adjustment to achieve optimal peak purity, adequate resolution between MG and 7OH-MG, and suitable retention times. Different mobile phase compositions were systematically evaluated to obtain satisfactory chromatographic separation and system performance.

#### 3.5.2. Preparation of Kratom Leaf Extract Samples

The kratom leaf extract was dissolved in a suitable solvent to obtain a sample concentration of approximately 1–2 mg/mL. The solution was sonicated for approximately 15 min or until complete dissolution was achieved. The resulting solution was filtered through a 0.22 µm syringe filter prior to HPLC-DAD analysis.

#### 3.5.3. Preparation of MG and 7OH-MG Standard Solutions

Reference standards of MG and 7OH-MG were individually dissolved in methanol to obtain stock solutions at concentrations of approximately 1–2 mg/mL. The stock solutions of both standards were then mixed and serially diluted to prepare mixed standard solutions at five concentration levels, with MG concentrations ranging from 2 to 80 µg/mL and 7OH-MG concentrations ranging from 0.5 to 20 µg/mL. All standard solutions were filtered through a 0.22 µm syringe filter prior to HPLC-DAD analysis.

#### 3.5.4. Method Development

Optimization of the HPLC-DAD separation conditions was carried out by varying the type, ratio, and pH of the mobile phase, based on conditions reported in previous studies. The chromatographic conditions were compared and evaluated according to the acceptance criteria specified in USP43–NF38, including resolution (≥2.0), symmetry factor (0.8–1.5), and number of theoretical plates (>2000) [23]. Peak purity, retention time, and overall chromatographic performance were also considered. Chromatograms obtained from mixed standard solutions of MG and 7OH-MG were compared with those from kratom leaf extract samples to ensure adequate separation and specificity.

#### 3.5.5. Method Validation

Quantitative analysis of MG and 7OH-MG in kratom leaf extract samples was validated using the optimized HPLC-DAD conditions in accordance with ICH guideline Q2(R1) (ICH, 2005 [25]). The validation parameters included system suitability, specificity, linearity and range, accuracy, precision, LOD, and LOQ.

System suitability was evaluated by analyzing the mixed standard solution of MG and 7OH-MG five consecutive times using the developed HPLC-DAD method. The evaluated parameters included resolution, retention time, symmetry factor, and number of theoretical plates.

Specificity was assessed by spiking the mixed standard solution of MG and 7OH-MG into the kratom leaf extract solution (1–2 mg/mL). The spiked samples were analyzed in triplicate under the developed HPLC-DAD conditions. Specificity was evaluated based on retention time, resolution, and peak purity by comparing chromatograms of the extract, the mixed standards, and the spiked samples.

Linearity was evaluated by analyzing five concentration levels of the mixed standard solutions of MG (2–80 µg/mL) and 7OH-MG (0.5–20 µg/mL). Each concentration level was analyzed in triplicate. Calibration curves were constructed by plotting peak area versus concentration, and linear regression analysis was performed to obtain the regression equation and coefficient of determination (R^2^).

Accuracy was assessed using the recovery study by spiking mixed standard solutions of MG and 7OH-MG at three concentration levels (75%, 100%, and 125%) into the kratom leaf extract samples. Each level was analyzed in triplicate. The percentage recovery (%recovery) was calculated.

Precision was evaluated in terms of repeatability (intra-day precision) and intermediate precision (inter-day precision). For repeatability (intra-day precision), mixed standard solutions of MG and 7OH-MG at three concentration levels were spiked into kratom leaf extract samples and analyzed three times within the same day. The relative standard deviation (%RSD) of the measured concentrations was calculated. For intermediate precision (inter-day precision), the same spiked samples at three concentration levels were analyzed three times per day over three consecutive days. The %RSD and HorRat values were calculated using the same acceptance criteria as those applied for intra-day precision.

LOD and LOQ were estimated from the calibration curves of the mixed standard solutions of MG and 7OH-MG.

Robustness was evaluated by increasing the column temperature from 20 to 25 °C. A mixed standard solution of MG and 7OH-MG was analyzed in triplicate under each condition, and system suitability parameters were assessed. No significant changes in chromatographic performance were observed, confirming the robustness of the method to minor temperature variations.

### 3.6. Quantitative Determination of MG and 7OH-MG in Kratom Leaf Extract

After successful method validation, the developed HPLC-DAD method was applied to quantify MG and 7OH-MG in kratom leaf extract samples. The extract solutions were prepared at concentrations of approximately 1–2 mg/mL and analyzed under the validated chromatographic conditions. Each sample was analyzed in triplicate. The concentrations of MG and 7OH-MG were calculated and expressed as mg/g of extract.

### 3.7. Statistical Analysis

All validation experiments were performed in triplicate, and the results were expressed as percentage recovery (%recovery) or mean ± standard deviation (mean ± SD), as appropriate. Method validation parameters were evaluated in accordance with the ICH guideline Q2(R1) and the AOAC guidelines (2016) for the quantitative analysis of MG and 7OH-MG in kratom leaf extract samples [21,22]. Statistical differences among groups were assessed using one-way analysis of variance (one-way ANOVA). Differences were considered statistically significant at *p* < 0.05. Statistical analyses were performed using SPSS software (version 23.0; IBM Corp., Armonk, NY, USA).

## 4. Conclusions

This study provides a validated analytical approach for the simultaneous quantification of MG and 7OH-MG and applies it to elucidate phytochemical variability in kratom. The results demonstrate that intrinsic botanical factors, particularly geographical origin and plant tissue type, are major determinants of alkaloid composition, whereas differences between ethanol-based ultrasound- and microwave-assisted extraction were comparatively limited. Distinct tissue-specific distribution patterns were observed, with shoots containing higher MG concentrations despite lower extraction yields, while 7OH-MG was detected exclusively in leaves. These findings confirm that extraction yield alone does not reflect alkaloid composition and highlight substantial compositional variation within kratom raw materials. Taken together, this study demonstrates that intrinsic botanical factors, particularly geographical origin and plant tissue type, are more influential determinants of kratom alkaloid composition than extraction technique under standardized ethanol-based conditions. The validated HPLC–DAD method and comparative phytochemical dataset generated in this work provide a practical framework for quality standardization, raw material selection, and future development of kratom-derived pharmaceutical and nutraceutical products. However, a limitation of the present study is that all kratom samples were collected exclusively from Thailand. Therefore, caution should be exercised when extrapolating these findings to kratom originating from other geographical regions, such as Indonesia or Malaysia, where environmental conditions, cultivation practices, and genetic backgrounds may differ and potentially influence alkaloid composition. Overall, this work contributes to the chemical characterization and compositional standardization of kratom as a bioactive plant material and provides a basis for quality-oriented evaluation of kratom-derived products.

## Figures and Tables

**Table 1:** Chromatographic conditions 1–6 evaluated during method development for the separation of 7-hydroxymitragynine (7OH-MG) and mitragynine (MG) using HPLC-DAD.

HPLC Condition	1	2	3	4	5	6
Column	Luna^®^ 5 µm C18(2) 100 Å LC column (250 × 4.6 mm)					
Column temperature	25 ± 1 °C	20 ± 1 °C				
Mobile phase	A: 5.0 mM ammonium bicarbonate buffer (pH 9.50) B: Acetonitrile	A: 0.1% formic acid in water B: Acetonitrile	A: 0.1% trifluoroacetic acid (TFA) in water B: Acetonitrile			
Gradient condition	Time (min); A (%):B (%) 0 min; 20:80 2 min; 20:80 5 min; 30:70 10 min; 60:40 15 min; 80:20 20 min; 80:20 25 min; 20:80	Time (min); A (%):B (%) 0 min; 70:30 25 min; 30:70 35 min; 30:70 40 min; 0:100 50 min; 0:100 55 min; 70:30 60 min; 70:30	Time (min); A (%):B (%) 0 min; 70:30 25 min; 30:70 35 min; 30:70 40 min; 0:100 50 min; 0:100 55 min; 70:30 60 min; 70:30	Time (min); A (%):B (%) 0 min; 80:20 15 min; 30:70 20 min; 30:70 22.5 min; 80:20 25 min; 80:20	Time (min); A (%):B (%) 0 min; 80:20 15 min; 30:70 20 min; 30:70 22.5 min; 80:20 25 min; 80:20	Time (min); A (%):B (%) 0.0 min; 80:20 15.0 min; 30:70 16.0 min; 0:100 20.0 min; 0:100 22.5 min; 80:20 25.0 min; 80:20
Flow rate	1.0 mL/min	1.5 mL/min	1.5 mL/min	1.0 mL/min	1.5 mL/min	1.5 mL/min
UV detector	226 nm (4 nm bandwidth)					
Injection	10 µL	5 µL				
Run time	25 min	60 min	60 min	25 min	25 min	25 min

**Table 2:** Chromatographic performance parameters, including retention time, peak area, symmetry factor, theoretical plates, peak purity, and resolution, for 7-hydroxymitragynine (7OH-MG) and mitragynine (MG) in mixed standard solutions (MixSTD) and spiked kratom leaf extracts analyzed under gradient conditions 4 and 5 using a mobile phase consisting of 0.1% trifluoroacetic acid and acetonitrile.

Sample	Retention Time (min)		Peak Area (mAU·s)		Symmetry Factor		Theoretical Plates (N)		Peak Purity		Resolution (Rs)
	7OH-MG	MG	7OH-MG	MG	7OH-MG	MG	7OH-MG	MG	7OH-MG	MG	
Gradient condition 4 (flow rate 1 mL/min)											
MixSTD	8.240	11.573	807.37860	2002.84277	1.22	1.15	36,641	76,237	999.968	999.966	19.58
Spiked extract	8.242	11.571	366.41470	1755.63721	1.96	1.18	36,429	69,464	996.061	999.859	1.94
Gradient condition 5 (flow rate 1.5 mL/min)											
MixSTD	6.330	9.647	546.05353	1376.22791	1.15	1.11	26,107	60,585	999.906	999.957	21.13
Spiked extract	6.340	9.656	240.07149	1138.97803	1.27	1.16	27,110	59,295	999.293	999.809	4.51

Resolution (Rs) represents the chromatographic separation between the adjacent peaks of 7-hydroxymitragynine (7OH-MG) and mitragynine (MG), with 7OH-MG eluting prior to MG, as calculated by the chromatographic software (version C.01.10). Peak purity was evaluated using DAD spectral analysis.

**Table 3:** System suitability results obtained from nine consecutive injections of a mixed standard solution containing mitragynine (MG, 48.0 µg/mL) and 7-hydroxymitragynine (7OH-MG, 12.0 µg/mL) analyzed under the optimized HPLC–DAD conditions.

Injection No.	Peak Area (mAU·s)		Symmetry Factor		Theoretical Plates (N)		Resolution (Rs)
	7OH-MG	MG	7OH-MG	MG	7OH-MG	MG	
1	90.39440	995.51880	1.03	1.01	22,979	53,635	19.70
2	90.62756	998.47443	1.08	1.02	22,519	53,275	19.52
3	87.87316	972.88324	1.04	1.00	23,087	51,737	19.47
4	87.38880	1000.09839	1.04	1.01	23,007	53,004	19.60
5	87.54860	969.79865	1.05	1.00	23,109	53,168	19.61
6	87.96274	1002.12653	1.04	1.00	23,090	54,183	19.73
7	88.08106	971.16394	1.04	1.00	23,105	53,522	19.65
8	84.77478	968.95959	1.03	0.99	22,465	51,782	19.37
9	88.58807	1001.09827	1.03	0.98	22,459	51,350	19.23
Mean	88.14	986.68	−	−	−	−	−
SD	1.73	15.30	−	−	−	−	−
%RSD	1.96	1.55	−	−	−	−	−

Resolution (Rs) represents the chromatographic separation between the adjacent peaks of 7-hydroxymitragynine (7OH-MG) and mitragynine (MG), with 7OH-MG eluting prior to MG, as calculated by the chromatographic software.

**Table 4:** Specificity evaluation of the HPLC–DAD method for the determination of mitragynine (MG, 32 µg/mL) and 7-hydroxymitragynine (7OH-MG, 8 µg/mL) in mixed standard solutions (MixSTD), kratom extracts (1.026 mg/mL), and spiked samples.

Sample	Injection No.	Retention Time (min)		Peak Purity		Resolution (Rs)
		7OH-MG	MG	7OH-MG	MG	
MixSTD	1	6.37	9.67	999.66	999.96	19.70
	2	6.37	9.67	999.98	999.95	19.60
	3	6.37	9.67	999.98	999.96	19.37
Extract	1	6.36	9.76	999.95	999.97	3.79
	2	6.37	9.76	999.87	999.82	3.71
	3	6.36	9.75	999.89	999.95	3.72
Spiked sample	1	6.38	9.73	999.85	999.85	3.84
	2	6.39	9.74	999.71	999.65	3.72
	3	6.39	9.75	999.87	999.72	3.84
	Mean ± SD	6.37 ± 0.01	9.72 ± 0.04	999.79 ± 0.19	999.87 ± 0.12	-

Acceptance criteria: Resolution (Rs) ≥ 0.95; peak purity > 999.00. Resolution (Rs) represents the chromatographic separation between the adjacent peaks of 7-hydroxymitragynine (7OH-MG) and mitragynine (MG), with 7OH-MG eluting prior to MG, as calculated by the chromatographic software. Peak purity was evaluated using DAD spectral analysis.

**Table 5:** Accuracy results expressed as percentage recovery (%recovery) and relative standard deviation (%RSD) for MG and 7OH-MG in spiked kratom extract samples (*n* = 3).

Analyte	Spiking Level (%)	%Recovery (Mean ± SD)	%RSD
MG	75	109.15 ± 0.25	0.23
	100	109.47 ± 0.08	0.07
	125	107.68 ± 0.17	0.16
7OH-MG	75	108.24 ± 0.17	0.16
	100	103.10 ± 0.36	0.35
	125	98.28 ± 0.61	0.62

Acceptance criteria: %recovery 80–110%; %RSD ≤ 2.0%.

**Table 6:** Intra-day and inter-day precision results expressed as %RSD and HorRat values for MG and 7OH-MG determined in spiked kratom leaf extract samples at three concentration levels.

Precision	Analyte	Spiking Level (%)	%RSD	HorRat Value
Intra-day precision	MG	75	0.30	0.03
		100	0.17	0.02
		125	0.31	0.03
	7OH-MG	75	0.61	0.06
		100	1.54	0.16
		125	1.26	0.13
Inter-day precision	MG	75	0.12	0.01
		100	0.13	0.01
		125	0.32	0.03
	7OH-MG	75	1.82	0.19
		100	1.62	0.17
		125	1.62	0.17

Acceptance criteria for all spiking levels: %RSD ≤ 2.0; HorRat ≤ 2.0.

**Table 7:** Limit of detection (LOD) and limit of quantitation (LOQ) of MG and 7OH-MG determined by the HPLC–DAD method.

Parameter	MG (µg/mL)	7OH-MG (µg/mL)
LOD	1.56	0.64
LOQ	4.72	1.94

**Table 8:** Robustness evaluation of the HPLC–DAD method for the determination of mitragynine (MG) and 7-hydroxymitragynine (7OH-MG) under nominal column temperature (20 °C) and increased column temperature (+5 °C, 25 °C).

Injection No.	Retention Time (min)		Peak Area (mAU·s)		Symmetry Factor		Theoretical Plates (N)		Peak Purity		Resolution (Rs)
	7OH-MG	MG	7OH-MG	MG	7OH-MG	MG	7OH-MG	MG	7OH-MG	MG	
Nominal condition (20 °C)											
1	6.37	9.67	87.54860	971.16394	1.05	1.00	23,109	53,522	999.96	999.96	19.70
2	6.37	9.67	87.96274	972.88324	1.04	1.00	23,190	51,737	999.98	999.95	19.60
3	6.37	9.67	87.87316	969.79865	1.04	1.00	23,087	53,168	999.98	999.96	19.37
Mean	6.37	9.67	87.79483	971.28194	1.04	1.00	23,129	52,809	999.97	999.96	19.56
SD	0.00	0.00	0.22	1.55	−	−	−	−	−	−	−
%RSD	0.00	0.00	0.25	0.16	−	−	−	−	−	−	−
+5 °C Robustness condition (25 °C)											
1	6.37	9.67	87.54858	971.16389	1.05	1.00	23,109	53,525	999.96	999.98	19.72
2	6.37	9.67	87.95272	972.88322	1.04	1.00	23,185	51,729	999.97	999.98	19.68
3	6.37	9.67	87.88300	969.79860	1.04	1.00	23,087	53,165	999.96	999.92	19.30
Mean	6.37	9.67	87.79477	971.28190	1.04	1.00	23,127	52,806	999.96	999.96	19.57
SD	0.00	0.00	0.22	1.55	−	−	−	−	−	−	−
%RSD	0.00	0.00	0.25	0.16	−	−	−	−	−	−	−

A mixed standard solution containing mitragynine (MG, 48.0 µg/mL) and 7-hydroxymitragynine (7OH-MG, 12.0 µg/mL) was injected in triplicate under each condition. Values are presented as individual measurements and mean ± SD. Resolution (Rs) represents the chromatographic separation between adjacent peaks of 7OH-MG and MG, with 7OH-MG eluting prior to MG, as calculated by the chromatographic software. Peak purity was evaluated using a diode array detector (DAD) spectral analysis. SD and %RSD were calculated for retention time and peak area only. Robustness was assessed by comparing chromatographic parameters under nominal column temperature (20 °C) and elevated temperature (25 °C) in accordance with ICH Q2(R1) acceptance criteria.

**Table 9:** Summary of method validation parameters for the determination of MG and 7OH-MG in kratom leaf extracts using the HPLC–DAD method.

Test	Method	Sample	Acceptance Criteria	Result
System suitability	Nine replicate injections of mixed standard solution (MixSTD) at a single concentration	MixSTD (MG 48.0 µg/mL, 7OH-MG 12.0 µg/mL)	%RSD of peak area ≤ 2.0% Symmetry factor ≤ 2.0 Resolution ≥ 2.0 Theoretical plates (N) > 2000	Pass
Specificity	Triplicate injections of MixSTD and spiked samples	Extract (1.0 mg/mL) MixSTD (single concentration) Spiked sample (2.0 mg/mL extract: MixSTD, 1:1 *v*/*v*; MG 64 µg/mL, 7OH-MG 16 µg/mL)	Comparable retention times Resolution ≥ 2.0 Peak purity > 999.00	Pass
Accuracy	Triplicate injections of spiked samples	Spiked samples at 75%, 100%, and 125% of MixSTD	Recovery 80–110% %RSD ≤ 2.0%	Pass
Precision – Intra-day – Inter-day	– Six replicate injections within the same day – Triplicate injections on three different days	Spiked samples at 75%, 100%, and 125% of MixSTD	%RSD ≤ 2.0% HorRat ≤ 2.0	Pass
Linearity and range	Six concentration levels, triplicate injections, performed in three independent runs	MixSTD (MG: 8–80 µg/mL; 7OH-MG: 2–20 µg/mL)	Coefficient of determination (R^2^) ≥ 0.9990	Pass
Sensitivity – LOD – LOQ	Analysis of diluted standard solutions under optimized conditions	Diluted MixSTD	LOD = 3.3 × (SD of intercept/slope) LOQ = 10 × (SD of intercept/slope)	Pass
Robustness	Triplicate injections of mixed standard solution under nominal column temperature (20 °C) and increased temperature (+5 °C, 25 °C)	MixSTD (MG 48.0 µg/mL, 7OH-MG 12.0 µg/mL)	%RSD of retention time and peak area ≤ 2.0% Symmetry factor ≤ 2.0 Theoretical plates (N) ≥ 80% of nominal condition Resolution (Rs) ≥ 2.0	Pass

**Table 10:** Extraction yield (% yield), mitragynine (MG) content, and 7-hydroxymitragynine (7OH-MG) content of kratom leaf extracts from different vein types and geographical regions obtained using ultrasound-assisted extraction (UAE) and microwave-assisted extraction (MAE).

Extract Code	Kratom Type	Region	Extraction Method	Particle Size (µm)	Yield (%)	MG Content (µg/mg Extract)	7OH-MG Content (µg/mg Extract)
H-N-UAE	Red vein (Hang Kang)	North	UAE	97.30	20.49 ± 0.57 ^II^	48.61 ± 0.18 ^E^	ND
H-N-MAE	Red vein (Hang Kang)	North	MAE	97.30	19.97 ± 0.34 ^II^	48.20 ± 0.09 ^D^	ND
R-N-UAE	Red vein	North	UAE	86.30	25.96 ± 0.37 ^I^	57.57 ± 0.02 ^H^	1.91 ± 0.03 ^a^
R-S-UAE	Red vein	South	UAE	112.22	18.40 ± 0.22 ^III^	46.88 ± 0.08 ^B^	2.71 ± 0.01 ^f^
R-N-MAE	Red vein	North	MAE	86.30	25.11 ± 0.58 ^I^	57.20 ± 0.15 ^G^	1.92 ± 0.01 ^a^
R-S-MAE	Red vein	South	MAE	112.22	17.91 ± 0.22 ^III^	46.64 ± 0.06 ^B^	2.49 ± 0.01 ^d^
G-N-UAE	Green vein	North	UAE	78.32	25.76 ± 0.09 ^I^	58.07 ± 0.06 ^I^	2.59 ± 0.04 ^e^
G-S-UAE	Green vein	South	UAE	106.66	18.43 ± 0.14 ^III^	47.39 ± 0.06 ^C^	2.33 ± 0.01 ^c^
G-N-MAE	Green vein	North	MAE	78.32	25.38 ± 0.44 ^I^	56.35 ± 0.08 ^F^	2.06 ± 0.03 ^b^
G-S-MAE	Green vein	South	MAE	106.66	17.30 ± 0.72 ^III^	46.14 ± 0.11 ^A^	2.52 ± 0.00 ^d^

Data are expressed as mean ± SD (n = 3). Different superscript letters (A–I for MG, a–f for 7OH-MG, and I–III for % yield) indicate statistically significant differences among samples (*p* < 0.05). Statistical analysis was performed using one-way analysis of variance (ANOVA) with SPSS version 23. ND: not detected.

**Table 11:** Physical characteristics and representative images of kratom leaf extracts obtained from different vein types and geographical regions using ultrasonic-assisted extraction (UAE) and microwave-assisted extraction (MAE). The corresponding extraction yield, MG content, and 7OH-MG content are presented in Table 10.

Kratom Type	Region	Extraction Method (Extract Code)	
		UAE	MAE
Red vein (Hang Kang)	North	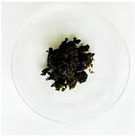 (H-N-UAE)	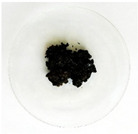 (H-N-MAE)
Red vein	North	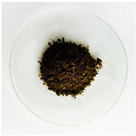 (R-N-UAE)	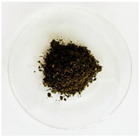 (R-N-MAE)
Red vein	South	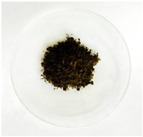 (R-S-UAE)	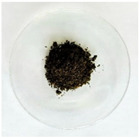 (R-S-MAE)
Green vein	North	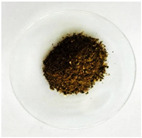 (G-N-UAE)	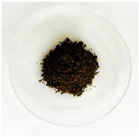 (G-N-MAE)
Green vein	South	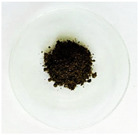 (G-S-UAE)	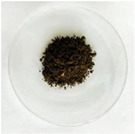 (G-S-MAE)

**Table 12:** Extraction yield (% yield), mitragynine (MG) content, and 7-hydroxymitragynine (7OH-MG) content of extracts obtained from shoots and leaves of green-veined and red-veined kratom from the central region of Thailand (Nakhon Pathom, NP) using ultrasound-assisted extraction (UAE).

Extract Code	Kratom Type	Plant Part	Yield (%)	MG Content (µg/mg Extract)	7OH-MG Content (µg/mg Extract)
R-NP(S)	Red vein	Shoot	14.77 ± 0.46 ^A^	74.53 ± 0.08 ^c^	ND
R-NP(L)	Red vein	Leaf	19.77 ± 1.16 ^C^	70.32 ± 0.05 ^d^	1.22 ± 0.04
G-NP(S)	Green vein	Shoot	15.65 ± 0.22 ^A^	79.75 ± 0.07 ^a^	ND
G-NP(L)	Green vein	Leaf	18.42 ± 0.61 ^B^	75.22 ± 0.15 ^b^	0.91 ± 0.03

Values are expressed as mean ± standard deviation (*n* = 3). Different uppercase letters (A–C) within the same column indicate statistically significant differences in % yield, whereas different lowercase letters (a–d) indicate statistically significant differences in MG content (*p* < 0.05). Statistical analysis was performed using one-way ANOVA with SPSS software (version 23). ND = not detected.

**Table 13:** Physical characteristics of dried kratom materials, powders, and extracts prepared from shoots and leaves of green-veined and red-veined kratom from the central region of Thailand using ultrasound-assisted extraction (UAE). The corresponding extraction yield, MG content, and 7OH-MG content are presented in Table 12.

Extract Code	Kratom Type	Plant Part	Dried Plant Material	Powdered Plant Material	Extract
R-NP(S)	Red vein kratom	Shoot	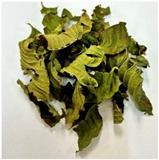	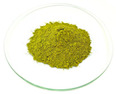	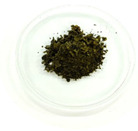
R-NP(L)	Red vein kratom	Leaf	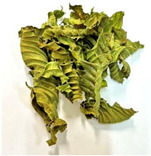	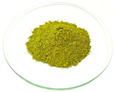	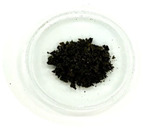
G-NP(S)	Green vein kratom	Shoot	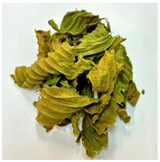	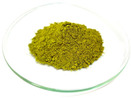	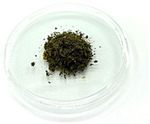
G-NP(L)	Green vein kratom	Leaf	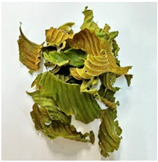	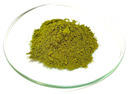	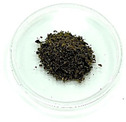

## Data Availability

The data presented in this study are available from the corresponding author upon reasonable request.

## Abbreviations

The following abbreviations are used in this manuscript:

7OH-MG	7-Hydroxymitragynine
ASE	Accelerated solvent extraction
HPLC–DAD	High-performance liquid chromatography coupled with diode-array detection
HPLC–UV	High-performance liquid chromatography coupled with ultraviolet detection
HorRat	Horwitz ratio
LC–MS/MS	Liquid chromatography–tandem mass spectrometry
LOD	Limit of detection
LOQ	Limit of quantitation
MAE	Microwave-assisted extraction
MG	Mitragynine
MixSTD	Mixed standard solutions
R^2^	Coefficient of determination
Rs	Chromatographic resolution
SFE-CO_2_	Supercritical carbon dioxide extraction
TFA	Trifluoroacetic acid
UAE	Ultrasound-assisted extraction
UHPLC–HRMS	Ultra-high-performance liquid chromatography coupled with high-resolution mass spectrometry
